# Exploring physicians’ prescribing behavior in patients with multiple sclerosis in Saudi Arabia: a sequential explanatory mixed-methods

**DOI:** 10.1186/s12883-023-03184-9

**Published:** 2023-03-31

**Authors:** Hussain Abdulrahman Al-Omar, Nada Alsowaida, Lama Aldosari, Ahmed Mayet, Reem Bunyan, Mohammed Aljumah

**Affiliations:** 1 Department of Clinical Pharmacy, College of Pharmacy, King Saud University, P.O. Box: 2457, Riyadh, 11451 Saudi Arabia; 2 Health Technology Assessment Unit (HTAU), College of Pharmacy, King Saud University, P.O. Box: 2457, Riyadh, 11451 Saudi Arabia; 3 Department of Pharmacy Services, King Saud University Medical City, P.O. Box: 2457, Riyadh, 11472 Saudi Arabia; 4 Department of Pharmacy, Maternity and Children Hospital, Ministry of Health, Al Kharj, 16278 Saudi Arabia; 5 Department of Neurology, Neurosciences Center, King Fahd Specialist Hospital, Dammam, 32253 Saudi Arabia; 6 Department of Neurology, King Fahad Medical City, Ministry of Health, Riyadh, 11525 Saudi Arabia

**Keywords:** Multiple sclerosis, Prescribing behavior, Physicians, Saudi Arabia, Mixed-methods

## Abstract

**Background:**

Multiple sclerosis (MS) is the most common disabling neurological disease in young adults worldwide with majority of patients manifest symptoms between 20 and 40 years of age. The aims of this study are to explore physicians’ perspectives, views, and behaviors in diagnosing and treating patients with MS in Saudi Arabia and investigate the prescribing pattern of disease-modifying therapies (DMTs).

**Methods:**

A sequential explanatory mixed-method approach was used to achieve the study objectives. The quantitative arm of the study consisted of patient data extracted from the Saudi MS registry from 2015 to 2018. The qualitative study consisted of in-depth semi-structured interviews with physicians using a validated interview topic guide comprising 28 open-ended questions.

**Results:**

We extracted data of 2,507 patients from 20 different hospitals across Saudi Arabia. Patients’ mean age was 34 ± 10 years; two-thirds (n = 1,668) were female. 92% (n = 2,292) had relapsing-remitting multiple sclerosis, and 5% (n = 126) had secondary-progressive multiple sclerosis. In general, patients with MS received at least one drug as the DMT or DMTs and corticosteroids for those with relapse. Qualitatively, nine physicians agreed to participate in the interviews. Of them, five (55%) were male and four were female from different regions. Thematic analysis yielded three main themes: practice, views, and challenges.

**Conclusions:**

The prevalence of MS in Saudi Arabia is raising but is still much lower than that reported in the Gulf region. A national MS guideline is needed to streamline diagnosis and treatment criteria, avoid any delay in treatment, and guide physicians who provide care for patients with MS.

**Supplementary Information:**

The online version contains supplementary material available at 10.1186/s12883-023-03184-9.

## Background

Multiple sclerosis (MS) is an immune-mediated inflammatory disease that destroys myelin and axons of the central nervous system to varying degrees and causes significant physical disability in some patients over time and has a high impact on families, health care system, and society [1]. The range and severity of MS manifestations in an individual at a particular time reflects the extent of lesions, their location, the severity of tissue damage, and the rate of accumulation of demyelinated lesions [2]. It is the most common disabling neurological disease in young adults worldwide [3]. Almost 70% of patients manifest symptoms between 20 and 40 years of age, with a mean age of onset of approximately 30 years. There is a clear gender difference, with women being more frequently affected than men [ratio, 2.5:1] [4]. The worldwide incidence and prevalence of MS are highly variable. Genetic and geographical factors are strongly associated with MS [2]. An increase in the prevalence of MS has been noted in the Gulf region; the estimated prevalence in the Gulf region was reported to be 31–55 MS per 100,000 population [5], placing the region in the low-medium risk zones according to the global MS prevalence scale [6]. In Saudi Arabia, the projected prevalence is estimated to be 61.95/100,000 Saudi individuals [7].

Current MS treatments consist primarily of disease-modifying therapies (DMTs) to minimize relapses and delay disease progression in conjunction with symptomatic treatment and supportive care. Although DMTs have a favorable impact on relapsing-remitting MS (RRMS) and suppress disease activity [8], they have limited benefits in progressive forms of MS, especially PMS and secondary-progressive MS (SPMS), in which neurological disability continues to worsen over time [2]. Drugs available for progressive forms of MS are indicated for cases showing clinical/radiological activity. Ocrelizumab is an approved drug for treating primary progressive MS, while siponimod and cladribine are approved for treating relapsing forms of MS. The approval of medications has led to a substantial change in the approach to the treatment of MS [8, 9].

Despite the effectiveness of DMTs, physicians are facing immense challenges in prescribing these medications [9, 10]. These challenges include selecting an appropriate DMT to treat MS patients, choosing between escalation or early intensive treatment [11], defining response and treatment failure, and deciding which agent to switch to in cases of inadequate response [9].

The treatment of MS in Saudi Arabia has not been well studied. Only a few studies have reported some aspects of MS [7, 12]. Furthermore, there is no qualitative research addressing the local practice of physicians’ prescribing behavior when it comes to MS diagnosis, therapy, and challenges. To improve the management of MS in Saudi Arabia, it is crucial to have detailed data on disease clinical characteristics, treatment patterns and yet unmet medical needs. Therefore, our study aimed to explore physicians’ perspectives, views, and behaviors in diagnosing and treating patients with MS in Saudi Arabia and investigate the prescribing pattern of DMTs.

## Methods

We used a sequential explanatory mixed-method approach to identify the current status of patients with MS in Saudi Arabia based on registry data; generate an in-depth understanding of physicians’ views, behaviors, and prescribing patterns for patients with MS; and identify areas need and challenges to improve patients’ outcomes. This information cannot be cultivated using quantitative or qualitative methods.

The quantitative study consisted of patient data extracted from the Saudi MS national registry from 2015 to 2018. The registry was launched in Saudi Arabia in 2015, to collect data on MS epidemiology, characteristics of MS patients, and therapies for those patients with confirmed MS diagnosis according to the 2010 McDonald criteria. The registry includes data obtained from 20 tertiary care hospitals in Saudi Arabia that reflect a representative sample of treating hospitals. The registry managed by a steering committee (consisting of three main investigators) and a scientific committee (consisting of 10 neurologists from different sites across the different regions in Saudi Arabia. A detailed description of the registry can be found elsewhere [13].

Data were extracted for a single time-point for each patient (first entry in the registry). Patients’ extracted data included demographics, gender, age, region, marital status, education level, occupation, family history, and comorbid disease. Disease-related information included diagnosis, expanded disability status scale (EDSS) score, age at the first MS attack, symptoms at the first MS attack, number of relapses in the first year, number of hospitalizations, affected organs (cortical, brain stem, cerebral or spinal cord), source of referral, type of referral, time between the onset of the disease and diagnosis (months), treatment for relapse, and DMT. At the time of data collection, the following DMTs were included in the MS registry: teriflunomoide, interferons including interferon beta-1a and interferon beta-1b, glatiramer acetate, fingolimod, alemtuzumab, mitoxantrone, rituximab, dimethyl fumarate.

The qualitative study consisted of in-depth semi-structured interviews using a validated interview topic guide comprising 28 open-ended questions. The interview topic guide was developed and reviewed by the authors to ensure that the study objectives were met. Interview questions fell into four domains: physicians’ views about MS, level of disease activity and prognosis, drug-related concerns and individual patient profile, and diagnosis and treatment guideline recommendations (Supplementary Table 1). Interviews were conducted using the purposeful sampling technique. The recruited physicians were of different gender, ages, regions, areas of practice, and educational backgrounds to maximize variation. We invited 19 general neurologists and MS specialists to participate in this study. All participants who agreed to take part in the qualitative interviews were informed about the quantitative data analysis and the objectives of the qualitative study and they were requested to provide their views, prespectives and feedback in light the results from data collected in period from 2015 to 2018. All interviews were conducted between May and June 2021 and were digitally recorded, transcribed verbatim to text format, and then imported to NVivo^®^ Version 11 (QSR International, Doncaster, Australia) to facilitate data coding and sorting.

For quantitative data, variables were analyzed using descriptive statistics and results were presented in the form of frequencies, percentages, means, and standard deviations, as appropriate. All analyses were performed using IBM^®^SPSS^®^ software (version 24.0; IBM Corporation, Armonk, NY). For qualitative data, the six-step approach of thematic analysis proposed by Braun et al. (2006) was used to analyze participants’ views [14]. The analysis was performed by ND and LM independently, and then the findings were discussed with HA to agree on the final themes and sub-themes. Then, the findings were presented using a table containing the main themes, sub-themes, and supporting quotes.

## Results

### MS registry quantiative data

We extracted data of 2,507 patients from 20 different hospitals across Saudi Arabia from year 2015 to 2018 (Table 1). Patients’ mean age was 34 ± 10 years; two-thirds (n = 1,668) were female. More than half (n = 1,436) of them had university level or higher education, and one-third (n = 798 patients) had high school level education. Approximately 13% (n = 315) had a family history of MS in all regions, ranging from 8.1% in the southern region to 16.2% in the central region. Two-thirds of patients with MS (n = 1,573) were initially observed and examined by family physicians. Subsequently, they were referred to MS specialists or speciality centers. 92% (n = 2,292) had RRMS, and 5% (n = 126) had SPMS.

**Table 1:** Baseline patients characteristics from the Saudi MS registry

Patients characteristics		Western Region n (SD)	Central Region n (SD)	Eastern Region n (SD)	Southern Region n (SD)	Northern Region n (SD)	Total n (SD)
**Mean age ± (SD)**		34.08 (10.21)	32.59 (9.25)	33.46 (9.32)	33.29 (9.54)	33.98 (9.58)	33.41 (9.69)
**Mean age of 1st attach ± (SD)**		28.5 (9.39)	27.29 (8.22)	26.93 (8.82)	27.54 (8.81)	29.5 (8.6)	27.81 (8.85)
**Mean number of previous relapse ± (SD)**		1.2 (1.45)	1.05 (1.47)	0.7 (1.09)	1.41 (1.53)	1.33 (1.13)	1.1 (1.42)
**Mean EDSS Score ± (SD)**		1.78 (2.27)	1.77 (1.95)	2.28 (1.9)	2.1 (2.39)	1.51 (2.11)	1.86 (2.12)
**Mean time between disease onset and diagnosis (months) ± (SD)**		9.05 (25.50)	11.36 (24.48)	11.96 (27.41)	12.85 (27.54)	13.40 (32.82)	10.8 (25.97)
**Patients characteristics**		**n (%)**	**n (%)**	**n (%)**	**n (%)**	**n (%)**	**Total n (%)**
**Male**		325 (32.9)	287 (33.3)	125 (33.3)	59 (34.1)	43 (39.4)	839 (33.5)
**Female**		663 (67)	575 (66.7)	250 (66.7)	114 (65.9)	66 (60.6)	1668 (66.5)
**Family history of MS**		106 (10.8)	136 (16.2)	47 (13.1)	14 (8.1)	12 (11.2)	315 (12.8)
**Affected sibling**		44 (5)	59 (8.1)	22 (7.7)	7 (4.4)	2 (2)	134 (6.3)
**Disease course**		**n (%)**	**n (%)**	**n (%)**	**n (%)**	**n (%)**	**Total**
**RRMS**		1037 (90.5)	857 (93.7)	307 (95.3)	52 (98.1)	39 (97.5)	2292 (92.6)
**SPMS**		89 (7.7)	28 (3.1)	7 (2.1)	1 (1.9)	1 (2.5)	126 (5.1)
**PPMS**		14 (1.2)	27 (2.9)	4 (1.2)	0 (0)	0 (0)	45 (1.8)
**PRMS**		6 (0.5)	2 (0.2)	4 (1.2)	0 (0)	0 (0)	12 (0.5)

MS: Multiple Sclerosis; RRMS: Relapsing Remitting Multiple Sclerosis; PPMS: Primary Progressive Multiple Sclerosis; SPMS: Secondary Progressive Multiple Sclerosis; PRMS: Progressive Relapsing Multiple Sclerosis EDSS: Expanded Disability Status Scale; SD: Standard Deviation.

In general, patients with MS received at least one drug, such as the DMT or DMT and corticosteroids, for those with relapse. During disease exacerbation, almost all patients with MS (n = 2,172, 99%) received corticosteroid therapy, mostly intravenous methylprednisolone. For DMTs, interfrons preparations and fingolimod were the most frequently prescibed drugs among other DMTs (Table 2).

**Table 2:** Treatment details from the Saudi MS registry by disease course

Medications	RRMS n (%)	SPMS n (%)	PPMS n (%)	PRMS n (%)	Total n (%)
Relapse	2003 (92)	137 (6.3)	35 (1.6)	12 (0.6)	2177 (47.2)
Corticosteroids	1998 (99.7)	127 (100)	35 (100)	12 (100)	2172 (99.7)
**DMTs**	**2296 (92.6)**	**131 (5.3)**	**35 (1.4)**	**18 (0.7)**	**2480 (52.8** ^*^ **)**
Teriflunomoide	101(4.4)	6 (4.58)	0 (0.0)	1 (5.56)	108 (4.35)
Avonex (interferon beta-1a)	466 (20.3)	26 (19.85)	7 (17.5)	2 (11.11)	501 (20.16)
Betaserone (interferon beta-1b)	562 (24.48)	29 (22.14)	13 (32.5)	6 (33.33)	610 (24.55)
Glatiramer acetate	10 (0.44)	1 (0.76)	0 (0.0)	1 (5.56)	12 (0.48)
Fingolimod	325 (14.16)	18 (13.74)	5 (12.5)	2 (11.11)	350 (14.08)
Alemtuzumab	6 (0.26)	0 (0.0)	0 (0.0)	0 (0.0)	6 (0.24)
Mitoxantrone	6 (0.26)	0 (0.0)	0 (0.0)	0 (0.0)	6 (0.24)
Rebif (interferon beta-1a)	507 (22.08)	36 (27.48)	7 (17.5)	4 (22.22)	554 (22.29)
Rituximab	21 (0.91)	1 (0.76)	1 (2.50)	0 (0.0)	23 (0.93)
Dimethyl fumarate	36 (1.57)	0 (0.0)	0 (0.0)	0 (0.0)	36 (1.45)
Natalizumab	256 (11.15)	14 (10.69)	2 (5.0)	2 (11.11)	274 (11.03)

*five patients switched to another DMTs; MS: Multiple Sclerosis; DMTs: Disease-Modifying Therapies; RRMS: Relapsing Remitting Multiple Sclerosis; PPMS: Primary Progressive Multiple Sclerosis; SPMS: Secondary Progressive Multiple Sclerosis; PRMS: Progressive Relapsing Multiple Sclerosis.

### Qualitative interviews findings

Nine physicians agreed to participate in the semi-structured interviews. All of them were familiar with the MS disease registry, as they were active participants in the collection of registry data. Among them, five (55%) were male and four were female from different regions. At specialties level, two participants (22%) were gneral neurolgoists, while the rest were MS specialists. The average interview time ranged from 35 to 45 min. Table 3 shows participants’ region of practice, sector, and the number of patients with active and highly active MS in their practice regions.

**Table 3:** Reported disease activity encountered by participating physicians

Interviewee	Physician Specialty	Disease Activity			Region
		Reported % of Active MS Cases	Reported % of Highly Active MS Cases	Sector	
P1	GN	30–35%	20%	Government	Western
P2	MSS	80%	40%	Government	Central
P3	GN	20–25%	10%	Government	Western
P4	MSS	80%	50%	Government	Eastern
P5	MSS	30–40%	30–40%	Government	Southern
P6	MSS	60%	20%	Semi-Government	Eastern
P7	MSS	20–25%	2–5%	Government	Central
P8	MSS	80-85%.	10–15%	Government	Eastern
P9	MSS	80%	5–10%	Private	Western

GN: General Neurologist; MSS: Multiple Sclerosis Specialist.

The qualitative data thematic analysis yielded three main themes: practice, views, and challenges. The main themes were further divided into 16 sub-themes namely: time from symptoms to diagnosis; time from diagnosis to treatment; escalation of therapy; utilization of new treatment; definition of active disease; definition of highly active disease; acceptable disease activity; causes of lack of standardization; minimization of lack of standardization; disease related challenges; facility related infrastructure; physicians’ knowledge; referral and disease nature; patient awareness about MS; logistics; and patients’ expectations and anticipation. All these themes and sub-themes are discussed in Supplementary Table 2. Overall, no variations were identified in the captured views, perceptions and insights between general neurologists versus MS specialists.

### Practice findgings

In the practice theme, most interviewees (general neurologists/MS specialists) agreed that the time from symptom appearance to a final diagnosis of the disease widely varies as one interviewee said, “I would think it takes from one month to three months on average.” The delay depended on how the patients reported their symptoms, at the time of the physician visit, and the performance of additional investigation. The time from diagnosis to treatment depended on the patient’s acceptance of the disease; as stated by the interviewee, sometimes patients hesitate to see the neurologist: “They do not want to start treatment immediately; they want to think; they want to get a second opinion.” Conversation with a patient is sometimes challenging. “The biggest challenge for me is the patient themselves. You have [patients] who do not have a clue of what are they saying, and they just give me what they think is the right thing. Or, sometimes there are patients who are too anxious about any medication. And, the number two [challenge] is the patient’s expectations. Many patients expect that they [will] get better with the treatment. So, they expect improvement of their symptoms; a resolution of their deficit.” There are many controversies surrounding the disease and initiating medications. For treatment escalation, most agreed to escalate therapy depending on disease progression and symptoms. Disease activity depends on the radiological progression and clinical failure. For highly active disease, most straight-selected high efficacy DMTs without going through the steps. New treatment options have been increasingly added to the market recently and are expected to improve with advances in technology and drug discovery. Although variety offers the luxury of choice, it adds another challenge for treating physicians. To best utilize the newer treatments, most of the participating physicians agreed that all DMTs are options for MS that should be available for all patients. The treating physicians must select the appropriate drug. The patients should be part of the shared decision-making process in selecting a DMT, as some patients care about convenience, whereas others care more about safety. An interviewee said, “It’s a difficult choice; it’s good that we have many options, but at the same time, it is challenging to choose appropriate medication for [the] appropriate patient. But, if you notice, some of the new medications that are in the market, there [is a] group of medication[s] [that has] almost [the] same mechanism of action; you find three or four approved [ones], but [you] have almost [the] same mechanism of action. I do not see a point from rushing to a new medication. If we have [a] good option available with a good experience with it, because I am not fine with once [a] medication [is on] the market, I will go and try it. If it’s needed, I will try it, especially [if] all of the medications are just disease-modifying [therapies]; they are not [the] cure if the medication is a cure for the patient; this is a different story, but if we have good options that we have good experience with medication why to jump and try something totally new?”.

Regarding the use of available MS guidelines in practice, most interviewees agreed that they were aware of the recommendations of different international guidelines [15–19]. Along with their experience in the field, they are individualizing the therapy as appropriate without major deviation from international consensus. The guidelines usually lack updated evidence, and most used personal experience. An interviewee said: “You have different guidelines, European, American. So, the problem with these guidelines, they usually lag behind evidence. So, if you’re someone who reads the evidence every year and you’re in the field, you go to conferences; you’re probably going to be updated even before the guidelines. For treatment, we go by European, North American, and the most recent evidence.” Another interviewee highlighted that the guidelines do not answer everything, and efforts on networking with other consultants and reviewing the literature take place in some cases. “We do not have [a] clear guideline or clear approach because we have [a] different school, a lot of controversies in the definition. When we escalate, we do not have [a] frame line. When I have to escalate, we say I will start early, but how early? Most [experts] will say in the first 6 months, but we do not have clear definitions to be honest with you.”

### Views findings

Regarding the views theme, most viewed an active disease when a patient has either relapses or recent relapses; recent changes on magnetic resonance imaging (MRI), e.g., T2 lesions, new T2 lesions, and new enhancing lesions; or progression of symptoms or recent progression of symptoms. For highly active disease, controversies on the definition exist, as one interviewee stated, “It is similar to active disease with no clear definition as highly active disease. It depends on the number of lesions, locations of the lesions, and [whether] the patient [is] already on disease-modifying therapy; however, still, the patient develops relapse, attacks, and receives pulse therapy more than once a year.”

Most of the participants disagreed on the definition of inactive disease; some considered having a lesion on MRI without symptoms as inactive disease, but others were cautious using this definition. Instead, they suggested that each patient’s status should be reviewed as MS guidelines and definitions frequently change.

Most physicians believed that there should be a specialized referral center to manage patients with MS. General physicians in peripheral hospitals should not treat MS patients with high-cost drugs or high-risk patients. A multidisciplinary care approach was suggested to oversee patients with MS and evaluate outcomes. One interviewee said, “I think having a center of excellence for MS management and MS care [is important]. People are moving toward multidisciplinary care and also managing MS patients. It’s not just simply seeing a neurologist, having other sub-specialties involved.”

### Challenges findings

The challenges highlighted by interviewees were logistics and accessibility to MRI machines, especially in smaller cities and regions. Another challenge highlighted by participants is the lack of speialized MS speicilists or general neurologists in remote areas in diffeent regions. One interviewee commented, “You wish to have a machine, an easy access.” In addition, drug availability is a major constraint for patients and neurologists. Sometimes the neurologists diagnose patients with a subtype of MS and prescribe the drug, which is suitable for the patient, but the drug is unavailable in the health care system. The other drug availability-related challenge for patients is their insurance. The insurance companies deny their drugs; it may be because they are not on the insurance plan’s “formulary.” One of the specialists stated, “The most important challenge is the insurance; most of [the] time, it affects the patient’s journey a lot. Sometimes I refer the patient to other [hospitals] because of the insurance. But, hopefully the guideline that we are working on will solve this issue, because most of the time they say that there is no guideline or its conflicting. But, I’m sure after approving our guideline, we will make difference.” The establishment of local guidelines for MS can solve this issue by including several options for DMT.

## Discussion

To understand how physicians diagnose and treat MS patients in Saudi Arabia, we conducted a mixed-methods research to analyze data obtained from the Saudi MS disease registry for the period from 2015 to 2018 and disccuss the views and perceptions captured from nine physicians through semi-structured interviews from different regions in Saudi Arabia to set up the scene for the forthcoming results that will be generated from from the recently launched MS registry in Saudi Arabia. Our study has identified several substantial challenges in various aspects of care in Saudi Arabia that are associated with MS diagnosis and treatment, including time for the first diagnosis, access to care, infrastructure, acceptance of guidelines recommendations, and access to new DMTs.

Our study found that the time from the onset of the symptom to final diagnosis ranges from 1 month to 6 months. The variation in the reported time may be explained by referral delay, image acquisition, timing of patient presentation, scheduling appointments with physicians, availability of imaging facilities, and quality of MRI imaging. The MRI acquisition parameters can substantially affect the detection of focal MS pathology. Therefore, neurologists recommend that standardized brain and spinal cord MRI protocols are necessary for diagnosis [20]. For follow-up and follow-up, MRI spine should be requested if there is evidence of spinal cord symptoms. Some variation was also observed in the estimated time to diagnose MS between the qualitative (1 to 6 months) and quantitative (11 months) analyses. This difference was due to the questionnaire data collection in the quantitative study compared to the real-time data extracted from the MS registry.

In this study, the use of corticosteroids by MS patients who are already receiving DMT may be indicative of relapse or exacerbation of the patient’s condition and that the treatment with DMT may be suboptimal for these patients. The response of MS patients to a DMT may not be immediate, so a relapse may occur when using a DMT [21]. Although the cross-sectional nature of this study does not allow for definite conclusions, it can be speculated that failure to change DMT following multiple corticosteroid treatments may indicate clinical inertia in which treatment did not change despite apparent therapy failure. During the study period, most MS patients were taking interferons instead of more effective drugs, e.g., natalizumab or fingolimod, which are recommended for MS patients with an insufficient response to first-line treatments or those with high disease activity. In summary, the prescription patterns of corticosteroid therapy and interferon-beta were similar across the regions. During our interviews, physicians reported a shift in the MS treatment approach and early escalation of therapy recently, which was not fully captured by our registry. Unlike other DMTs, the administration of natalizumab or fingolimod in MS patients was found to be associated with a lower annual rate of relapse; therefore, the need for corticosteroids is much less [22]. Although natalizumab and fingolimod are highly efficacious, the use of these DMTs requires careful patient monitoring due to the higher risk of severe adverse events [23].

Among the interviewees, there was no consensus on the definition of disease activity [active [24] and highly active [25, 26]] because these terms are not well-defined in the guidelines; it depended mainly on the clinical presentation, number of lesions on brain MRI, and frequency of patient relapse. For example, if naïve patients presented with mild symptoms that is less than three lesions on MRI, they were considered to have active disease. Highly active patients were defined as those who presented with more severe symptoms with numerous lesions on MRI with frequent relapses or those who received several pulse therapies in a year or had already been treated with DMT. This point of view was brought into discussion by our participants possibly due to the fact that there is still a lack of imaging and biological markers that would distinguish MS phenotypes and prognosticate the disease course on an individual patient’s level, creating a pressing need for large collaborative consensusing on MS definition.

In general, most neurologists found MS treatment is challenging, particularly in deciding on the appropriate DMTs form efficacy, safety, availability, and cost prespectives. The DMT selection also dependeds on various factors: the sub-type of MS, disease activity, age, gender, affordability, route of administration, degree of disability, comorbidity, EDSS score, and disability score. A qualitative study highlighted that when selecting DMTs, patients’ preferences concerning adverse effects, mode of administration, and frequency of administration should be considered [27]. Our research noted the unavailability of DMTs in treating facilities and the difficulty in obtaining DMTs through insurers due to poor formulary management of the hospitals. Additionally, most neurologists discouraged family physicians from prescribing DMTs, because they do not have enough experience and in-depth knowledge in managing MS; therefore, training more physicians to become neurologists or MS specialists is a priority in the country to ensure that patients with MS will be managed and monitored optimally and according to their disease course and stage. Other challenges in managing MS were: no single diagnostic test for MS but that depends on criteria, delayed referral to neurologists, and inappropriate management by family physicians before patients were seen by neurologists. Most neurologists agreed to escalate the selected DMTs without going through the steps in patients with highly active diseases. Therefore, all DMT options for MS should be available to all patients. Early initiation of the selection of high-efficacy DMTs decreases secondary progression of the disease and controls relapses [28, 29].

Some neurologists preferred an aggressive (accelerated) treatment approach for highly active disease, which was primarly driven by their personal experience. Controversy exists in selecting new DMT options, and careful examination was recommended in determining appropriate therapy. Most neurologists believed that developing a local guideline would be helpful in standardizing practice; all participants were willing to participate in such situation. A Saudi guideline for MS can address local experience, guidance, and logistic support for general practitioners. This may also resolve some of the challenges related to timely access to MS care at diagnosis.

This study has several strengths. The mixed-methods approach helped not only to identify the extent of the problem but also to explore and explain the behavior and actions of general neurologists and MS specialists who practice in Saudi Arabia. From a methodological perspective, the use of data derived from the MS registry instead of other data sources allowed the use of a large patient sample size. Multiple measures were used to ensure the trustworthiness of the qualitative study, maintaining credibility, transferability, dependability, and confirmability, thus building the strength of the study in general. These measures included approaching physicians with different specialties to maximize variation, gathering background knowledge on MS, using an interview topic guide, ensuring the consistency of the transcripts by involving several independent authors, and presenting findings using appropriate quotations.

This study has some limitations. First, this study used data from the registry that were captured up to 2018 and did not reflect recent changes in treatment regimens associated with recently approved drugs in Saudi Arabia. Second, few patients data were missing and wherever there were missing data, analysis was performed by excluding the missing data. Third, the lack of other similar qualitative or mixed-methods studies limited the comparison of our findings with other reported findings. Fourth, the qualitative findings may lack generalizability. However, a detailed description of the research method and data interpretation process, an in-depth presentation of the findings, and use of a mixed-methods approach holds promise for future generalizability.

## Conclusions

This study sheds the light on the practice, views, and challenges of general neurologists and MS specialists in the management of patients with MS in Saudi Arabia. The prevalence of MS in Saudi Arabia is raising but still much lower than that reported in the Gulf region. It is more prevalent among younger women with mostly RRMS, and patients receive corticosteroids and interferon-beta therapy. The study suggests that a national MS guideline is needed to streamline diagnosis and treatment criteria, avoid any treatment delay, and guide physicians who are providing care for patients with MS. Cooperative procurement of MS therapies is also needed because MS drugs are expensive and unavailable in most hospitals. With the shortage of MS specialists in Saudi Arabia, neurologists should be aware of patients’ disease experiences, treatment preferences, and support. This study’s results can be considered as baseline for future research to compare the impact of the introduction of a national MS guideline on patient care and physician prescribing behavior.

## Electronic supplementary material

Below is the link to the electronic supplementary material.

Supplementary Material 1

## Data Availability

All data generated or analysed during this study are included in this published article and its supplementary information files.
