# Supplementary material for: Exploring physicians’ prescribing behavior in patients with multiple sclerosis in Saudi Arabia: a sequential explanatory mixed-methods
Source: BMC Neurol. 2023 Mar 31;23:135. doi: 10.1186/s12883-023-03184-9 (PMC10064662; doi:10.1186/s12883-023-03184-9)
Supplement: Supplementary file 1 — Supplementary Material 1 [file 12883_2023_3184_MOESM1_ESM.docx]

**Supplementary Table 1. Interview questions**

MS: multiple sclerosis; DMTs: disease-modifying therapy.

| **Domain** | **Question** |
| --- | --- |
| **Disease views** | - In your view, how do you see MS as a disease? - From where do get most of your patient referral? - What is the usual time between symptoms appearance and establishing a diagnosis? - What is the usual time between diagnosis and starting patient treatment? - What could be the possible gaps or challenges in diagnosing patients with MS? |
| **Disease activity and prognosis** | - How would you define the term active disease? - How would you define the term highly active disease? - Do you use the term diagnosis of inactive disease, if yes, how would you define it? - From your own experience and knowledge, how would you identify patients with rapidly evolving, highly active disease? - What is/are the acceptable threshold of disease activity? - Based on your own practice, how would you monitor patient’s disease activity (subjective and objective)? How frequent? - In your opinion, what could be the possible gaps or challenges in determining patients’ disease activity and prognosis? |
| **Drug issues and individual patient profile** | - In your practice, what are the factors you may consider when you plan to start newly diagnosed MS patient on DMTs? - What are the possible challenges you may face when deciding to start a patient on DMTs? - How does MS sub-types affect your treatment choice? - When do you usually switch a patient from one DMTs to another within the same treatment line? - How would you define the term treatment failure? - When do you usually switch a patient from one DMTs to another between different treatment lines? - How do you manage the washout period when switching between DMTs? - In your practice, when would you consider escalation approach in therapy? - In your practice, how would you define a patient with partial response to therapy? - How would you manage a patient with a partial response to therapy? - In your practice, when do you decide to use off-label medications? - How would you ensure and manage optimal utilization of newly introduced therapies? |
| **Diagnosis/treatment guidelines recommendations** | - Which guideline(s) do you follow when you diagnose and/or treat patients with MS? Why? |
|  | - In your opinion, what is/are the possible causes of lack of standardization in guidelines recommendations? |
|  | - In your view, how can we minimize the effect of lack of standardization in guidelines recommendations in MS practice? |
| **Closing** | - Is there anything else that I have missed in this interview and you would like to add it? |

RRMS: Relapsing Remitting Multiple Sclerosis; PPMS: Primary Progressive Multiple Sclerosis; SPMS: Secondary Progressive Multiple Sclerosis; PRMS: Progressive Relapsing Multiple Sclerosis.

**Supplementary Table 2. Analysis findings from participants’ semi-structured interviews**

| **Theme** | **Sub-Theme** | **Supporting Quote** |
| --- | --- | --- |
| **Practice** | Time from symptoms to diagnosis | Well, from symptom onset to diagnosis, there is a wide variation because firstly, it depends on the patient, how he or she reports the symptoms, how soon they go to see a doctor and then according to the symptom. What the first doctor kind is, neurologist, ophthalmologists, or sometimes orthopedics or something like that. So, from symptom onset to diagnosis, could be like a week, or 10 days, up to months. The variation is mostly related to a neurologist because from diagnosis, usually the patient reach neurologist. |
|  |  | From onset until diagnosis, quite variable some of the patient. I would thinking it takes from one month to three months on average. |
|  |  | Starting of symptoms until visit, I can say it take two to three weeks, until they come for me. |
|  | Time from diagnosis to treatment | Let us say within a week, or sometimes there is a delay, I would say the delay generally, being a neurologist I want to say up to three months, maybe? Sometime there is hesitation from the neurologist side or sometime the hesitation from the patient's side. They're not sure, they do not want to start treatment, they want to think, they want to get a second opinion |
|  |  | Typically, if they have active MS, it takes half month to one month they get on treatment. |
|  | Escalation of therapy | Progression of the disease, basically clinical and radiological progression. Including failure |
|  |  | If there is a treatment failure, generally speaking, if there is treatment failure, you escalate. For highly active, we just go straight. We do not go step. |
|  |  | It is fairly straightforward. If they have any new relapse. My goal is no relapses, no MRI activities, and no progression. If they have any of those after adequate trial of therapy, depends on the drug mechanism of action, then I will escalate. |
|  | Utilization of new treatment | I think all DMT options for MS should be available for all patients and then you have to individualize it. Some of the therapies, convenience for patient some of the patient care about convenience, but other patient care more about safety. I am a believer in a shared decision-making and deciding a disease-modifying therapy, you have to include your patient in the decision-making. |
|  |  | It’s a difficult choice, it’s good that we have many options but at the same time it is challenging to choose appropriate medication for appropriate patient but if you notice some of the new medication that are in the market there are group of medication have almost same mechanism of action, you find three or four approved but have almost same mechanism of action. I do not see a point from rushing to a new medication If we have good option available with good experience with it because I am not fine with once medication in the market I will go and try it. If it’s needed, I will try it especially all of the medications are just disease modifying therapy they are not cure if the medication is a cure for the patient this is different story but if we have good options that we have good experience with medication why to jump and try something totally new. |
|  |  | I think there is room for all of these agents. At the same time, I think that having the guidelines set that we produced is extremely helpful to narrow the variation in practice. So, sometimes you will need highly efficacious medication for a patient, and then you make a decision, one versus the other because again, of choice, a patient can take for example, a daily pill, but they prefer an IV treatment that they take periodically. And so, for them, sometimes that is the factor that helps them make a decision one way or the other. The reason I say there is room for all of these drugs is because MS is such a heterogeneous disease and it's very different than other diseases. |
| **Views** | Definition of active disease | Active disease means that patient is having either relapses and recent relapses, recent changes in the MRI, and individually will indicate T2 lesions, new T2 lesions, and new enhancing lesions, or there is a progression of symptoms, recent progression of symptoms. |
|  |  | There is no definition for active disease in the guidelines that all the physicians will follow, it depends on the experience. On my experience the active disease depend firstly on the lesions in the MRI findings also the lesions location very important if it is in the infratentorial or cervical or spinal cord. Also, the enhancing lesion in my opinion will indicate active disease. Secondly, from the clinical presentation the numbness and vison considered as mild disease not active disease. On another hand if the patient develops ataxic gait or fall down or weakness in my opinion this indicate active disease. |
|  |  | To be honest with you, I do not have a fixed definition. I will call it active disease if there is any clinical relapse, either clinically or radiologically. Clinically presence of a relapse, radiologically presence of lesions in the brain MRI. But I am depending on the number of the lesions of the brain MRI. Either I call it active or highly active. |
|  | Definition of highly active disease | So based on clinical activity and radiological activity, a highly active will be a combination of both. |
|  |  | For me if there are three lesions and above, I would consider it highly active disease. Also, the severity of relapses, and the time between the relapses. For example, if the patient has had one relapse, then six weeks later had another relapse, with many enhancing lesions in the brain, this is, I would consider it, also highly active disease. |
|  |  | Highly active disease? It is similar to active disease with no clear definition as highly active disease. It depends on the number of lesions, locations of the lesions, and the patient already on disease-modifying therapy; however, still, the patient develops relapse, attacks, and receives pulse therapy more than once a year. |
|  | Acceptable disease activity | In my opinion, the active disease will be acceptable if the patient is not complaining from attacks. For example, If there is new lesion in the MRI or new enhancing lesion but the patient still the same no new symptoms, no complication and clinically stable in this case I will consider it as acceptable disease activity. |
|  |  | It is not every relapsing-remitting would have a complete resolution of that relapse. Having said that, so acceptable will be something that does not affect their functionality. Now that could also be very variable. Somebody, even just a resistance numbness in first season may affect. And it may interfere with their lifestyle or some it's like, it has to be a significant, you may have to say, is there a significant to any impairment that would affect them? |
|  |  | I have zero tolerance for disease activity. So I do not tolerate any single new lesions on MRI what matters more is the topography, right? Any single new lesion in the brain MRI, in a patient who has been otherwise stable, new lesion is very tiny or not enhanced. Then you might say, I will repeat the MRI after six months and then decide. But generally speak, I do not allow any disease activity. I mean otherwise stable patients, with no relapse, single definitive new lesion on a brain MRI, makes me uncomfortable. I might not change or escalate immediately but if I repeat the MRI in six months and there is another new lesion, and then I will escalate. But if more than one, for sure. I do not tolerate it. |
|  |  | I do not have any acceptable level. The objective is to control their disease and more and more now that is there is no minimum level of activity that you would accept because there are treatment options. |
|  | Causes of lack of standardization | Different school is one of other problem we face. A lot of controversy in approach, and starting with medications availability. |
|  |  | The problem with these guidelines, they usually lag behind evidence. So if you're someone who reads the evidence every year and you're in the field, you go to conferences, you're probably going to be updated even before the guidelines. |
|  |  | We do not have clear guideline or clear approach because we have different school, a lot of controversies in the definition. When we escalate, we do not have frame line. When I have to escalate, We say I will start early, but how early? Most of expert will say in the first six months, but we do not have clear definitions to be honest with you. |
|  |  | It's multifactorial. So there is a lot of things. Unfortunately, in the MS world, a lot of it especially lately, is heavily influenced by the pharmaceuticals. It's not necessarily in a bad way, meaning that only medication who are studied, are in the guidelines because will push for these medications and do phase three clinical trials. |
|  |  | There are other diseases that the patient population is more homogenous. In the case of MS, the disease is truly heterogeneous to the core and so sometimes some of these variations require equally variations in the way we treat them. |
|  | Minimization of lack of standardization | Having an easy access to MS neurologist not necessarily that all MS should be managed by MS neurologist but if the general neurologist think that he needs an immediate or urgent help of MS neurologist, I think we should have appropriate channel to communicate with MS neurologist. |
|  |  | I think having any center of excellence for MS management and MS care. I do not think a general neurologist in a peripheral hospital should have a decision to treat the patient with very expensive medication, highly risky. They do not have the experience; they do not have the knowledge. People are moving toward multidisciplinary care and also managing MS patients. It is not just simply seeing a neurologist, having other sub-specialties involved. I think there should be MS centers that are managing those patients, not to leave it for people who do not have the experience to manage. I think that is kind of the main challenge we have. The problem know we do not have enough specialists. We do not have enough centers that have the ability of treating and monitoring these patients. |
|  |  | I think the role for Multiple Sclerosis Foundation and what they are doing to standardize the guidelines around different region in Saudi Arabia. |
| **Challenges** | Disease related challenges | There is no single diagnostic test for MS. It depends on criteria. This is one of the challenges for diagnosis, because you have to have a good experience to be able to apply the criteria. A second, the presence of white matter lesion are present in many conditions, not only MS. Migraine, other conditions, to be able to differentiate this, this is MS or something mimicking MS, here is the challenge also. |
|  | Facility related infrastructure | You wish to have a machine, easy access. |
|  | Physicians’ knowledge | The main gap is knowledge, the practitioner's knowledge. |
|  |  | The private or the primary health care centers, they are not really well know the disease. So sometime we get a referral that is delayed. Like patient is followed for several months and then they are referred to us as a new symptoms and then we discovered by this patient could be an MS for a long period of time. So this is the major gap. |
|  | Referral / disease nature | It's referral problem. The patient will not come directly to neurologist. The patient will go around many clinics and the Symptoms of the multiple sclerosis are wide and variable so initially many miss it and last reserve usually is neurology. |
|  | Patients awareness about MS | Number one in patient recognition will be symptoms. We see sometimes patients come with numbness and they do not know this is an important thing, they ignore it so this is number one, causing delay. |
|  |  | I think sometimes the patient neglect or is not aware of the symptoms. So, the patient lacks awareness about the symptom. |
|  |  | The conversation we have with the patient is very challenging. It is too much for them and so I think the ability, the base knowledge of the patients about the disease itself. |
|  | Logistics | Drug availability is the most important challenge because sometimes you see a patient and you decide or you think that this patient or this drug is suitable for this particular patient and then the drug is not available. So these are the most important challenge actually. |
|  |  | The most important challenge is the insurance most of time it affects the patient’s journey a lot. Sometimes I refer the patient to other hospital because of the insurance. But hopefully the guideline that we are working on it will solve this issue because most of the time they say that there is no guideline or its conflicting but I am sure after approving our guideline we will make difference. |
|  | Patients’ expectations and anticipation | The biggest challenge for me is the patients themselves. You have either patients who do not have a clue of what are they saying and they just give me what they think is the right thing. Or, sometimes there are patients who are too anxious about any medication And the number two is the patient's expectations. A lot of the patients, they expect they get better with the treatment. So they expect improvement of their symptoms, a resolution of their deficit. |
|  |  | Some people they are really resistant. They are afraid from the immune suppression. |

MS, multiple sclerosis; DMT, disease-modifying therapy; MRI, magnetic resonance imaging.
